# A prospective study of magnetic resonance imaging and ultrasonography (MRI/US)-fusion targeted biopsy and concurrent systematic transperineal biopsy with the average of 18-cores to detect clinically significant prostate cancer

**DOI:** 10.1186/s12894-017-0310-7

**Published:** 2017-12-12

**Authors:** Yuji Hakozaki, Hisashi Matsushima, Jimpei Kumagai, Taro Murata, Tomoko Masuda, Yoko Hirai, Mai Oda, Nobuo Kawauchi, Munehiro Yokoyama, Yukio Homma

**Affiliations:** 1 Department of Urology, Tokyo Metropolitan Police Hospital, #4-22-1 Nakano, Nakano-ku, Tokyo, 164-0001 Japan; 2 Department of Radiology, Tokyo Metropolitan Police Hospital, Tokyo, Japan; 3 Department of Pathology, Tokyo Metropolitan Police Hospital, Tokyo, Japan; 4 Department of Urology, The University of Tokyo Graduate School of Medicine, Tokyo, Japan

**Keywords:** Clinically significant prostate cancer, Targeted biopsy, MRI/US fusion biopsy, PI-RADS version 2 score, Extended biopsy

## Abstract

**Background:**

This study compared the detection rates for clinically significant prostate cancer (CSPC) between magnetic resonance imaging and ultrasonography (MRI/US)-fusion-targeted biopsy (TB), systematic biopsy (SB) and combination of TB and SB.

**Methods:**

This prospective study evaluated simultaneous TB and SB for consecutive patients with suspicious lesions that were detected using pre-biopsy multiparametric MRI. A commercially available real-time virtual sonography system was used to perform the MRI/US-fusion TB with the transperineal technique. The prostate imaging reporting and data system version 2 (PI-RADS v2) was assigned to categorize the suspicious lesions.

**Results:**

A total of 177 patients were included in this study. The detection rate for CSPC was higher using SB, compared to TB (57.1% vs 48.0%, *p* = 0.0886). The detection rate for CSPC was higher using the combination of TB and SB, compared to only SB (63.3% vs 57.1%, *p* = 0.2324). Multivariate analysis revealed that PIRADS v2 category 4 and an age of <65 years were independent predictors for TB upgrading (vs. the SB result).

**Conclusions:**

PI-RADS v2 category 4 and an age of <65 years were predictive factors of upgrading the Gleason score by MRI/US-fusion TB. Thus, MRI/US-fusion TB may be appropriate for patients with those characteristics.

**Trial registration:**

This study was retrospectively registered at the University Hospital Medical Information Network (UMINID000025911) in Jan 30, 2017.

## Background

There is increasing evidence that multiparametric magnetic resonance imaging (mpMRI) of the prostate can improve the detection rates of clinically significant prostate cancer (CSPC) and prevent unnecessary biopsies. Extensive research has recently evaluated the efficacy of magnetic resonance imaging and ultrasonography (MRI/US)-fusion targeted biopsy (TB), although it remains unclear whether MRI/US-fusion TB could replace systematic biopsy (SB) [1–3].

We performed a prospective study to compare the diagnostic value of MRI/US-fusion TB and concurrent SB. Imaging-guided biopsy can be classified into three categories such as cognitive targeting without any technological guidance, targeting in the MRI gantry and real-time MRI/US-fusion guided biopsies. However, there is no visual feedback in the absence of technological guidance, and TB in the MRI gantry is time-consuming, so we adopted MRI/US-fusion guided biopsies using the transperineal technique in our hospital.

## Methods

### Study design

This was a prospective study approved by an institutional review board, and all participants provided written informed consent. All patients who presented to our hospital for prostate biopsy were recommended to undergo pre-biopsy multiparametric MRI (mpMRI) of the prostate. Patients with suspicious prostate lesions were subsequently recruited to undergo MRI/US-fusion TB and concurrent SB.

### Multiparametric MRI and biopsy methods

The mpMRI (Achieva 3.0 T TX: Philips Medical Systems, Best, Netherlands) was used to obtain T2-weighted fast spin-echo images in the transverse, sagittal and coronal planes, as well as diffusion-weighted (DW) images and dynamic-contrast enhanced (DCE) images. The detailed MRI parameters are shown in Table 1. For MRI/US-fusion TB, T2-weighted 3-D/sagittal images (70 slices with a thickness of 1 mm) were reconstructed. A commercially available real-time virtual sonography (RVS) system (Hitachi Medical Corporation, Tokyo, Japan) was used for the present study. The time resolution of the DCE images was 27.1 s. Suspicious lesions were marked using a circle on the axial images, and the corresponding sagittal images were automatically marked. Transperineal biopsies were performed with the patient under general or spinal anesthesia. For the TBs, a linear transrectal probe (HI VISION, Ascendus, Hitachi) and magnetic position sensors (3D Guidance Trakstar, Ascension) were used to obtain at least 2 cores from the targeted lesion [4]. After the TB, extended SB was performed in a prostate volume dependent manner along the parasagittal and far lateral lines with a 5 mm interval from 1 cm above the echo probe to the top of the prostate.

**Table 1:** The magnetic resonance imaging parameters

	Sequence type	Slice thickness, mm	No. of slices	TR, ms	TE, ms	b-values	Voxel size, mm
T2 sagittal	TSE	1	80	571	155	–	0.75 × 0.75 × 1
T2 coronal	TSE	3	20	4000	100	–	0.57 × 0.71 × 3
T2 axial	TSE	3	20	4000	100	–	0.57 × 0.72 × 3
DWI	SE-EPI	3	20	3773	84	2000 s/mm^2^	2.5 × 3.1 × 3
T1 DCE	FFE	0.85	85 × 6	6.6	3.4	–	0.85 × 0.85 × 0.85

*DWI* diffusion-weighted imaging, *DCE* dynamic contrast enhanced, *TR* repetition time, *TE* echo time, *TSE* turbo spin echo, *SE-EPI* spin echo-echo planar imaging, *FFE* fast field echo

### Clinically significant prostate cancer

We defined CSPC as cancers that did not fulfill all of the Epstein criteria for clinically insignificant cancer [5]: (i) prostate-specific antigen (PSA) density of <0.15, (ii) ≤50% involvement of any 1 core, (iii) a Gleason score of ≤3 + 3, and (iv) <3 positive biopsy cores. PSA value was not included.

### Statistical analysis

The Student t test and Pearson‘s chi-square test were used for comparing detection rates. Univariate and multivariate analyses were performed using the logistic regression model. All statistical analyses were performed using JMP® 12.2.0 (SAS Institute Inc., Cary, NC, USA).

### Image interpreting

One experienced radiologist (M.O.) scored the suspicious lesions according to the Prostate Imaging Reporting and Data System version 2 (PI-RADS v2) in a blinded fashion. The highest score of suspicious lesions was defined as the patient’s score.

### Upgrade

The highest Gleason score from the SB and TB specimens was considered the patient’s score. Pathology results were obtained for MRI/US-fusion TB and SB specimens, and cases were considered upgraded if one method provided a higher Gleason score, or if one method detected prostate cancer (PC) when the other did not detect PC.

## Results

Between January 2014 and March 2016, 177 consecutive patients were included in this study. Of these, 163 patients had one suspicious lesion on MRI and 14 patients had two. The patients’ profiles are shown in Table 2. The mean number of biopsied cores per patient was 3.8 by TB and 18.4 by SB. The median age and PSA were 68 (48–89) years and 7.42 (1.65–218) ng/mL, respectively. One patient was under active surveillance and had a Gleason score of 3 + 3.

**Table 2:** The patients overall clinical and histological characteristics

Patients, n	177
Age, years	68.3 (48–89)
Prebiopsy prostate-specific antigen level, ng/mL	10.9 (1.65–218)
Prostate volume, mL	42.4 (11–134)
Positive digital rectal examination result, %	51 (28.8)
Systematic cores per prostate, n	18.4 ± 2.1
Targeted cores per prostate, n	3.84 ± 0.4
Patients without prior biopsy, n	145
Patients with prior biopsy negative for cancer, n	31
Patients under active surveillance, n	1
Previous prostate-related treatment, n	0
Gleason score, n	
6 (3 + 3)	19
7 (3 + 4)	16
7 (4 + 3)	14
8 (4 + 4)	48
≥ 9 (4 + 5, 5 + 4, or 5 + 5)	19
Clinically significant prostate cancer, %	112 (63.3)

Data were reported as mean (range) or mean ± standard deviation

**Fig. 1:**
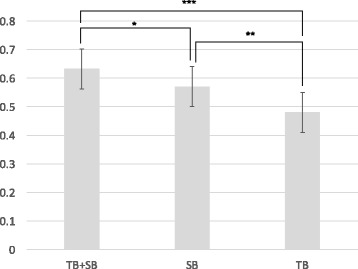
The detection rates for clinically significant prostate cancer with targeted biopsy (TB), systematic biopsy (SB), and the combination of both (TB + SB). *statistically insignificant with a *p* value of 0.2324. **statistically insignificant with a *p* value of 0.0886. ***statistically significant with a *p* value of 0.0039

A total of 116 patients (65.5%) were found to have PC. The detection rates of total prostate cancer were 49.7% for TB, 58.7% for SB and 65.5% for the combination of TB and SB (Table 3). The detection rates of CSPCs were 48.0% for TB, 57.1% for SB and 63.3% for the combination of TB and SB. The combination of SB and TB showed a statistically higher CSPC detection rate than that of TB (Fig. 1). The detection rate for CSPC was non-significantly higher for SB over TB and the combination over SB, since the *p*-values were >0.05. The detection rates of both total cancer and CSPCs were highest for the combination of SB and TB.

**Table 3:** The detection rates of prostate cancer and clinically significant prostate cancer

	TB + SB	SB	TB
Prostate cancer	116/177 (65.5%)	104/177 (58.7%)	88/177 (49.7%)
95%CI	58.2 to 72.1	51.4 to 65.7	42.4 to 57.0
CSPC	112/177 (63.3%)	101/177 (57.1%)	85/177 (48.0%)
95%CI	56.0 to 70.0	49.7 to 64.1	40.8 to 55.3

*TB* Targeted biopsies, *SB* Systematic biopsies, *CSPC* Clinically significant prostate cancer, *CI* Confidence interval

Regarding CSPC, approximately 10% (11/112) were not detected using SB. In contrast, 22%(25/112) were not detected using TB (Table 4).

**Table 4:** The correspondence table of the diagnosis between targeted biopsies and systematic biopsies

Diagnosis of TB	Diagnosis of SB		
	CSPC, n	Clinically insignificant Pca, n.	No cancer, n.
CSPC, no.	74	0	11
Clinically insignificant Pca, no.	2	0	1
No cancer, no.	25	3	61

*TB* targeted biopsy, *SB* Systematic biopsy, *Pca* Prostate cancer, *CSPC* Clinically significant prostate cancer

There was agreement between the Gleason scores for the TB and SB specimens in 62.7% (111/177) of all patients, and in 43.1% (50/116) of the patients with cancers (Table 5). 16 patients were diagnosed with prostate cancers using only TB or upgraded using TB (vs. the SB results). Univariate and multivariate regression analysis revealed that the PI-RADS v2 category 4 and an age of <65 years were independent predictors for TB-based upgrading (vs. the SB results) (Table 6). Receriver operating characteristics curve analysis indicated that PSA density > 0.17 was the strongest predictor of TB-based upgrading (area under curve = 0.582). However, univariate analysis showed PSA density was not a predictor for TB-based upgrading using this cut-off value.

**Table 5:** Agreement in the Gleason scores for the targeted and systematic biopsies

Histology of TB	Histology of SB					
	No cancer	3 + 3	3 + 4	4 + 3	4 + 4	≥9
No cancer	61	8	7	4	7	2
3 + 3	4	7	2	2	5	0
3 + 4	1	0	6	1	5	0
4 + 3	2	0	0	5	2	0
4 + 4	4	1	0	1	23	5
≥9	1	0	0	0	2	9

*TB* targeted biopsy, *SB* Systematic biopsy

**Table 6:** Univariate and multivariate analyses of upgrading predictions for targeted versus systematic biopsies

		Univariate analysis			Multivariate analysis		
		OR	95% CI	*p*-value	OR	95% CI	*p*-value
PI-RADS v2 category	5	1.14	0.37–3.45	0.8215	–	–	–
	4	4.24	1.40–12.8	0.0064^a^	4.33	1.46–14.6	0.0077^a^
PSAD >0.17 (yes vs. no)		2.71	0.74–9.91	0.1179	–	–	–
DRE (yes vs. no)		0.79	0.24–2.57	0.6910	–	–	–
Number of biopsies per PV ≥0.5 (yes vs. no)		1.77	0.62–5.11	0.2836	–	–	–
Age < 65 years (yes vs. no)		3.64	1.28–10.4	0.0111^a^	3.73	1.28–11.4	0.0165^a^
Repeat biopsy (yes vs. no)		1.05	0.28–3.92	0.9417	–	–	–

*OR* odds ratio, *CI* confidence interval, *PSAD* prostate-specific antigen density, *DRE* digital rectal examination, *PV* prostate volume

^a^statistically significant

## Discussion

PSA has been used for PC screening for over 20 years and TRUS-guided 10–12-core biopsy is still a standard diagnostic method as the number of biopsy cores is associated with improved PC detection rates. However, this approach can also detect indolent cancers. The US Preventive Service Task Force (USPSTF) recommended against PSA screening for PC to avoid over-diagnosis and over-treatment, which led to a decrease in the American use of the prostate biopsy. Recent studies have revealed that MRI/US-fusion TB provides higher detection rates of CSPC and is less invasive than PSA-based SB. However, it is unclear whether MRI/US-fusion TB can replace SB. In this study, we compared the detection rates of PC between MRI/US-fusion TB and concurrent extended SB with the transperineal technique. To the best of our knowledge, this is the first prospective study to compare MRI/US-fusion TB and extended SB with an average of 18 cores.

The results indicate that SB provided a higher detection rate of CSPC, compared to TB (57.1% vs 48.0%), and that only SB was able to diagnose 25 patients with CSPC. In contrast, some previous studies have indicated that TB provides high rates of PC detection [6, 7].

There are two reasons why SB provided higher rates of PC and CSPC detection in the present study. The first reason is that we performed transperineal biopsy along the parasagittal and far lateral lines with an interval of 5 mm and a prostate volume- dependent number of biopsy cores. This technique is similar to the template biopsy technique, and the PROMIS study revealed that the template biopsy technique was able to detect PC (119/452, 26.3%) in some cases that were missed by standard TRUS [8]. Hossack et al. reported that transperineal biopsy detected more anterior tumors than transrectal biopsy [9]. Although we did not perform template biopsy, our transperineal SB technique may have provided better PC detection, compared to standard 10–12-core random biopsy or transrectal biopsy. The second reason is that we used the Epstein criteria, which only define insignificant cancer to be present in cases with a Gleason score of 3 + 3, and only a few patients in the present study had insignificant cancers. Baco et al. used the same definition for significant/insignificant PC, and reported that 12-core random biopsy provided a higher detection rate, compared to MRI/TRUS-guided TB (49% vs 38%) [2].

We also detected CSPC using SB when TB provided negative results in 15.3% of the patients (27/177). It is possible that TB can fail to detect PC, as Ahmed et al. reported that CSPC was detected using template biopsy in 10.8% of patients (17/158) who had negative MRI findings [8]. Cash et al. reported that TB failure is the main cause of negative TB findings [10]. In this context, SB may detect PC in areas where MRI failed to do so or where TB did not effectively target the PC. This under-detection of PC could be caused by a lack of conspicuity on MRI because of image quality, which may result from image noise using very high b-values for DWI, or low temporal resolution for dynamic contrast-enhanced imaging. It could also result from misregistration and mistargeting when MRI correctly identifies suspicious areas [11, 12]. Histopathological results from radical prostatectomy are needed to address this issue, although only a few patients in the present study underwent radical prostatectomy.

Twelve patients were diagnosed with PC using TB but not using SB. To assess the utility of TB, we compared the Gleason scores from TB and SB respectively. There were 116 PC patients who were diagnosed using the combination of TB and SB. However, only 50 patients had the same Gleason scores for the TB and SB specimens. Furthermore, 16 patients were upgraded based on the TB results (vs. the SB results), and 34 patients were upgraded based on the SB results. A previous study revealed TB upgrading (vs. SB) in 22% of cases (43/198) [3], while the result was much lower in the present study (9.0%, 16/177). On the other hand, Muthigi et al. reported the upgrading rate for SB (vs. TB) as 23.9% of the cases (135/564) [13]. The upgrading rate for SB was a similar rate in the present study (28.2%, 50/177).

We assigned the PI-RADS v2 category to all lesions retrospectively and blindly. The PI-RADS v2 score provides a good prognostic value for PC and CSPCs [14, 15]. This scoring system was established in 2012 by the ESUR guidelines [16] and version 2 was updated in 2014. A consensus statement from the American Urological Association and the Society of Abdominal Radiology’s Prostate Cancer Disease-Focused Panel about the reporting system of MRI recommends the use of the PI-RADS v2 score and states that categories 3 to 5 should be targeted by image-guided biopsies [17].

To identify cases that would benefit from TB, we evaluated the factors that might predict TB upgrading. Univariate and multivariate analyses revealed that the PI-RADS v2 category 4 and an age of <65 years were independent predictive factors for upgrading for TB. The PI-RADS v2 category 5 was not a significant predictive factor. It is probably because the suspected lesions were larger than PI-RADS v2 category 4 lesions and CSPCs from category 5 lesions were easily detected using SB. It is unclear why an age of <65 years predicted upgrading for TB, although it is possible that interpreting MRI and targeting may be more difficult in older patients who have prostatic hypertrophy or prostatitis. The number of cores by SB was not a predicting factor of upgrading by TB, so increasing the number of SB cores should not be recommended to decrease upgrading of TB.

There are some limitations in our study. First, only one experienced radiologist assigned the PI-RADS v2 scores with the lesions. However, the inter-radiologist reproducibility of the PI-RADS v2 is good for experienced radiologists [18] and the PI-RADS v2 score has moderate inter-reader agreement. As the radiologist had over 10 year-career of interpreting PC imaging, it is likely that the results of this study are reliable. Second, only a few patients underwent radical prostatectomy. It will be important to collect more histological data from the radical prostatectomies with upgrading for TB. Third, the PI-RADS v2 categories were retrospectively assigned. Prospective scoring is necessary to assess the accuracy of the PI-RADS v2 category, although there was no bias for the urologists to perform the biopsies based on the PI-RADS v2 category in this study.

## Conclusion

In conclusion, the combination of TB and SB was a good tool to detect CSPCs. MRI/US-fusion TB might not be a suitable replacement for systematic transperineal biopsies. Nevertheless, 16 patients were upgraded based on TB findings. The PI-RADS v2 category 4 and an age of <65 years were predictive factors of upgrading for MRI/US-fusion TB, so MRI/US-fusion TB should be recommended in such patients. To the best of our knowledge, this is the first prospective study to compare MRI/US-fusion TB and extended SB with an average of 18 cores.

## Abbreviations

CSPC: Clinically significant prostate cancer
DCE: Dynamic-contrast enhanced
DW: Diffusion-weighted
mpMRI: Multiparametric MRI
MRI/US: Magnetic resonance imaging and ultrasonography
PC: Prostate cancer
PI-RADS v2: Prostate imaging reporting and data system version 2
RVS: Real-time virtual sonography
SB: Systematic biopsy
TB: Targeted biopsy
